# Paternal occupation and neuroblastoma: a case–control study based on cancer registry data for Great Britain 1962–1999

**DOI:** 10.1038/sj.bjc.6605504

**Published:** 2010-01-12

**Authors:** A MacCarthy, K J Bunch, N T Fear, J C King, T J Vincent, M F G Murphy

**Affiliations:** 1 Childhood Cancer Research Group, University of Oxford, Richards Building, Old Road Campus, Headington, Oxford OX3 7LG, UK; 2 Academic Centre for Defence Mental Health, King's College London, Institute of Psychiatry, Weston Education Centre, 10 Cutcombe Road, London SE5 9RJ, UK

**Keywords:** neuroblastoma, case–control study, paternal occupation

## Abstract

**Background:**

Neuroblastoma is the most common malignancy of infancy but little is known about the aetiological factors associated with the development of this tumour. A number of epidemiological studies have previously examined the risk associated with paternal occupational exposures but most have involved small numbers of cases. Here we present results from a large, population-based, case–control study of subjects diagnosed over a period of more than 30 years and recorded in the national registry of childhood tumours in Great Britain.

**Methods:**

A case–control study of paternal occupational data for 2920 cases of neuroblastoma, born and diagnosed in Great Britain between 1962 and 1999 and recorded in the National Registry of Childhood Tumours, and 2920 controls from the general population matched on sex, date of birth and birth registration district. Paternal occupations at birth, of the case or control child, were grouped by inferred exposure using an occupational exposure classification scheme. Conditional logistic regression was used to estimate odds ratios (ORs) and 95% confidence intervals (95% CI), for each of the 32 paternal occupational exposure groups.

**Results:**

Only paternal occupational exposure to leather was statistically significantly associated with neuroblastoma, OR=5.00 (95% CI 1.07–46.93). However, this association became non-significant on correction for multiple testing.

**Conclusion:**

Our findings do not support the hypothesis that paternal occupational exposure is an important aetiological factor for neuroblastoma.

Neuroblastoma is a tumour of the sympathetic nervous system (OMIM, 2009), and is the most common of the four embryonal tumours diagnosed in children. Tumours are most commonly found in the adrenal gland, the abdomen, the retroperitoneal area or the chest. In Britain, the tumour accounts for 6% of all cancers diagnosed in childhood (0–14 years), and about 90 cases, including ganglioneuroblastoma, are registered in the National Registry of Childhood Tumours (NRCT) each year. The majority of cases (86%) are diagnosed by 5 years of age, and they constitute 19% of all tumours diagnosed in infants under 1 year, being the most common single type of tumour in this age group (Stiller, 2007).

The early onset of this tumour may be related to aetiological factors that influence conception, pregnancy and early life, such as parental occupation. In view of this, we carried out a large, population-based, matched case–control study of data that relates to paternal occupational exposures at the time of birth of the case or control subject. A number of epidemiological studies have examined the possible association between paternal occupation and the occurrence of neuroblastoma in offspring, some examined paternal occupational exposures which are comparable to the 32 groups examined in our study (see Table 1). These included: case–control studies (Spitz and Johnson, 1985; Bunin *et al*, 1990; Wilkins and Hundley, 1990; Michaelis *et al*, 1996; Olshan *et al*, 1999; Kerr *et al*, 2000; Schüz *et al*, 2001; De Roos *et al*, 2001a, 2001b, 2001c; Pearce *et al*, 2006, 2007); a cohort study (Feychting *et al*, 2000)); and a population-based study by (Carlsen, 1996) which compared cases of neuroblastoma which present at different ages (see Table 2 for more details of each study). The results of these studies were inconclusive and the estimates reported were often based on small numbers.

## Patients and methods

### The selection of cases and controls

The population-based NRCT included 3219 cases of neuroblastoma born and diagnosed between 1962 and 1999, for 2920 cases (91%) a birth record was available. The remaining 299 cases, who were born or diagnosed overseas, adopted or not traced in the birth registers, were excluded from the study. The birth record for one control child per case matched on sex, date of birth (within 6 months), and birth registration sub-district was selected from the Office for National Statistics (ONS) or General Register Office in Scotland (GROS) birth registers.

Oxfordshire Research Ethics Committee (Oxfordshire REC C, Ref 07/Q1606/45) approved the use of the data reported in this study in 2007.

### Coding of occupational data and derivation of the occupational exposure groups

Paternal occupation (part of the public record of birth registrations) was abstracted verbatim from the birth records and coded according to the 1980 Classification of Occupations (Office of Population Censuses and Surveys, 1980). The occupations were coded ‘blind’ to case or control status, independently by two coders. A third coder then recoded occupations where there were discrepancies, occupations coded differently by all three were reviewed by a fourth coder. At the end of this process 71 occupations remained uncertain and were coded as ‘inadequately described.’ The 1980 codes were converted to the 1970 Classification of Occupations (Office of Population Censuses and Surveys, 1970) using an automated recoding scheme. The occupations were classified as to whether or not they involved definite contact (i.e., every day or at very high levels) with any of the 33 exposures listed in Table 1, (see (Fear *et al*, 1999) for more details). Some occupations were classified in more than one exposure group, for example ‘bus conductors’ were included in the following exposure groups: ‘exhaust fumes’, ‘inhaled hydrocarbons’ and ‘social contact’.

Some 261 birth records did not have a paternal occupation recorded and were coded as ‘missing data’ in all exposure groups. The 71 ‘inadequately described’ occupations (described above) and 18 occupations that could not be converted to the 1970 coding scheme, were considered in the analyses as ‘unexposed’ to all of the exposure groups.

### Analysis

Conditional logistic regression analyses (Breslow and Day, 1980) were carried out. As some of the exposure groups contained small numbers of exposed fathers, the analyses were carried out with LogXact 7 (2005) so that exact methods of statistical inference could be used. The exposure group ‘tobacco dust’ contained no case or control fathers. Odds ratios (ORs) and 95% confidence intervals (95% CI) were calculated for the remaining 32 exposure groups. Statistically significant results were defined as those where the *P*-value was <0.05. Any statistically significant ORs reflecting associations not previously reported in the literature were adjusted for multiple testing using the Bonferroni method (Bland and Altman, 1995).

## Results

The study population is described in terms of age at diagnosis and sex in Table 3. Table 1 shows our results for 32 exposure groups. One estimate of risk was statistically significant, that for exposure to ‘leather’ OR=5.00 (95% CI 1.07–46.93). However, after adjustment for multiple testing, this OR was no longer significant. The 12 case or control fathers in this exposure group had the following occupational titles: ‘clicker (shoe company)’, ‘shoe operative’ (*n*=2), ‘shoe worker’ (*n*=2), ‘leather cutter’, ‘leather worker’, ‘shoe repairer/journeyman’ (*n*=2), ‘tanner (fellmongers)’, ‘machine operator (brush factory)’, ‘boot and shoe operative (men's moulding)’.

## Discussion

Our findings for most exposure groups were unremarkable, with most ORs being around one. The only significantly raised risk – for the children of fathers exposed to leather – became non-statistically significant on correction for multiple testing. Estimated risks quoted in other epidemiological studies have varied in direction and magnitude. When comparing our results with those of other studies (listed in Table 2), it should be noted that in some of these studies information on occupational exposures has been collected from different sources, occupations and exposures were grouped using different classification schemes and in some studies the timing of the father's occupational exposure, with respect to the child's birth and diagnosis, was different from that in our study, which was ‘at birth’.

Many studies have found no significantly raised risks for the occupational exposure groups we studied. Below we discuss statistically significant findings for comparable groups of exposures from other studies.

### Agrochemicals

Our estimates for the varied group of occupational titles classified as having exposure to ‘agrochemicals’ were not directly comparable to the statistically significant findings from other studies quoted for specific exposure to ‘pesticides’, ‘herbicides’ or ‘insecticides’ (Michaelis *et al*, 1996; Schüz *et al*, 2001; Pearce *et al*, 2006).

### Electromagnetic fields

Spitz and Johnson (1985) reported a significantly raised risk for the offspring of men employed as ‘electricians, electric and electronics workers, linemen, utility employees, welders, electrical equipment salesmen and repairmen’: OR=2.13 (95% CI 1.05–4.35). This risk increased when the analysis was restricted to ‘electronics workers’ only, OR=11.75 (95% CI 1.40–98.55). Later studies (Bunin *et al*, 1990; Wilkins and Hundley, 1990; Olshan *et al*, 1999; Feychting *et al*, 2000; Kerr *et al*, 2000; De Roos *et al*, 2001b, 2001c; Pearce *et al*, 2007) reported non-significant estimates of risk (in both directions) for paternal occupational electromagnetic field exposures. The result of our study is in line with these later investigations.

### Metal and lead

Results for ‘Metal’ from earlier published studies were non-significant, (Wilkins and Hundley, 1990; Michaelis *et al*, 1996; Olshan *et al*, 1999; Kerr *et al*, 2000; De Roos *et al*, 2001a, 2001c). Carlsen (1996) did not quote a relative risk but examined the frequency of paternal job titles for neuroblastoma cases from two birth cohorts split by age at diagnosis, and noted that the percentage of fathers employed in the ‘manufacture of iron and metal structures’ was ‘rather high’. Kerr *et al* (2000) reported a statistically significant raised estimate of risk for lead: OR=2.4 (95% CI 1.2–4.8). Our findings for exposure to lead, based on larger numbers, did not support this finding.

### Coal dust

Kerr *et al* (2000) reported a raised risk for ‘coal soot’, OR=5.9 (95% CI 1.0–60.4, based on six case and two control fathers). Our estimate for ‘coal dust’ was much lower and not statistically significant.

### Interpreting the results of this study

Despite the size of our study, it has limited power for some exposure groups. However, these data represent all cases of neuroblastoma in a large national population-based registry, and the study is much larger than any previously reported. Exact methods of statistical inference were used to take account of the small numbers in some exposure groups.

Where there is an exposure prevalence in the controls of 3% for a particular exposure group, the study has more than 80% power to detect an OR of 1.5 in that group. Eleven of the exposure groups we examined: construction, electromagnetic fields, exhaust fumes, forces, hydrocarbons (inhaled and dermal), lead, metal, metal working (oil mists), solvents and social contact had at least this prevalence of exposure in the control group. Of these groups, paternal exposure to: ‘electromagnetic fields’, ‘metal’ and ‘lead’ had previously been reported as significantly associated with an increased risk of childhood neuroblastoma. There were no significant results reported for ‘construction’ or ‘solvents’. There were no comparable groups reported in other studies for the remaining six groups.

In our study the occupation was recorded before the diagnosis of neuroblastoma, eliminating the possibility of recall bias. Occupations were coded, and discrepancies resolved, ‘blind’ to case–control status and were allocated to exposure groups within an exposure classification scheme devised with the help of an occupational hygienist, which has been used in a number of other studies (Fear *et al*, 1998, 1999, 2007, 2009; McKinney *et al*, 2003; MacCarthy *et al*, 2009). It is possible that occupations may have been incorrectly assigned to an occupational code and hence assigned to an inappropriate exposure group. Given that the coding was undertaken blind to the status of each subject, any bias would be non-differential and this would usually be expected to bias the OR towards 1.00, obscuring a real relationship with the exposure group.

We do not have information about biomarkers of occupational exposure, and cannot adjust for the use of protective equipment. Unlike some earlier studies based on detailed information from questionnaires or interviews, we have no detailed information on the actual level or frequency of the occupational exposure. Also, occupational practices and exposures may have changed over the years covered by this study. Our study examines paternal occupation as recorded at the time of birth of the child, and occupations could have changed in the time between birth and diagnosis of neuroblastoma. As shown in Table 2, neuroblastoma is most often diagnosed at an early age, so this is less of a consideration than it might be for some other diagnostic groups.

Other possible risk factors may be acting as confounders in our study, these factors are reviewed in detail in the comprehensive literature review by Heck *et al* (2009). We thought that socio-economic status might be relevant to our study of occupational exposures; however, when we adjusted for the variable ‘manual’ or ‘non manual’ social class status of the father (a proxy for socio-economic status), our initial results were unchanged (data not shown). Heck *et al* (2009) reviewed data on socio-economic status in earlier studies and concluded that there was insufficient evidence to draw a conclusion about this as a risk factor for neuroblastoma. They did report that there was some evidence of a positive association with both maternal alcohol consumption and low birth weight, factors which could be indirectly correlated with socio-economic status, but on which we have no information.

## Conclusion

We have carried out the largest case–control study of paternal occupation and neuroblastoma possible in Britain. However, our study still has limited power for many of the defined exposure groups. Differences in study design also make it difficult to examine how our results relate to previous findings for occupational exposures. We have outlined a number of limitations in our study methods. Bearing these in mind, our findings for the exposure groups we examined do not support the hypothesis that paternal occupational exposure is an important aetiological factor for neuroblastoma.

## Figures and Tables

**Table 1:** Paternal occupational exposure groups for neuroblastoma cases and controls, odds ratios with 95% confidence intervals. Definite contact: i.e. contact everyday or at very high levels

**Exposure group**	**Case fathers (% exposed)**	**Control fathers (% exposed)**	**Informative pairs**	**Odds ratio**	**95% confidence interval**
1 Agriculture	58 (1.99)	63 (2.16)	102	0.89	0.59–1.34
2 Agrochemical	78 (2.67)	79 (2.71)	134	0.97	0.68–1.38
3 Animals	20 (0.68)	23 (0.79)	38	0.73	0.36–1.45
4 Ceramics/glass	3 (0.10)	7 (0.24)	9	0.29	0.03–1.50
5 Coal dust	27 (0.92)	22 (0.75)	43	1.39	0.73–2.70
6 Construction	204 (6.99)	210 (7.19)	362	1.01	0.82–1.25
7 Electromagnetic fields	168 (5.75)	183 (6.27)	304	0.90	0.71–1.13
8 Exhaust fumes	228 (7.81)	244 (8.36)	396	0.93	0.76–1.14
9 Fishing	4 (0.14)	6 (0.21)	10	0.67	0.14–2.81
10 Foodstuffs	95 (3.25)	86 (2.95)	166	1.05	0.76–1.44
11 Forces	117 (4.01)	106 (3.63)	198	1.15	0.86–1.54
12 Heat (prolonged exposure)	52 (1.78)	60 (2.05)	106	0.93	0.62–1.38
13 Hydrocarbons (inhaled)	399 (13.66)	430 (14.73)	607	0.95	0.80–1.11
14 Hydrocarbons (dermal)	196 (6.71)	224 (7.67)	342	0.86	0.69–1.07
15 Ionising radiation	5 (0.17)	3 (0.10)	7	1.33	0.23–9.10
16 Lead	83 (2.84)	94 (3.22)	160	0.86	0.62–1.19
17 Leather	10 (0.34)	2 (0.07)	12	5.00^*^	1.07–46.93
18 Medical/heath care	49 (1.68)	56 (1.92)	100	0.82	0.54–1.24
19 Metal	408 (13.97)	419 (14.35)	631	0.97	0.82–1.13
20 Metal acid mists	5 (0.17)	1 (0.03)	5	4.00	0.40–197.00
21 Metal fumes	38 (1.30)	40 (1.37)	73	1.03	0.63–1.67
22 Metal working (oil mists)	106 (3.63)	121 (4.14)	207	0.88	0.66–1.17
23 Mining	29 (0.99)	22 (0.75)	45	1.50	0.80–2.89
24 Paints	51 (1.75)	62 (2.12)	102	0.79	0.52–1.19
25 Paper production	2 (0.07)	1 (0.03)	3	2.00	0.10–118.00
26 Plastics	8 (0.27)	7 (0.24)	15	1.14	0.36–3.70
27 Printing	21 (0.72)	30 (1.03)	50	0.72	0.39–1.31
28 Rubber	4 (0.14)	8 (0.27)	11	0.57	0.12–2.25
29 Social contact	363 (12.43)	335 (11.47)	443	1.05	0.87–1.27
30 Solvents	79 (2.71)	97 (3.32)	148	0.80	0.57–1.13
31 Textile dust	34 (1.16)	31 (1.06)	58	1.32	0.76–2.32
32 Tobacco dust			0		
33 Wood dust	91 (3.12)	75 (2.57)	147	1.30	0.92–1.83

^*^Statistically significant (*P*<0.05) but non significant after correcting for multiple testing.

**Table 2:** Reports of studies of neuroblastoma and paternal occupational exposures

**Author, publication year**	**Neuroblastoma cases**			**Source of neuroblastoma cases**	**Source of paternal occupational data**	**Time of paternal occupational exposure**
Spitz and Johnson (1985)	157	Deaths aged 0–14 years	1964–1978	Bureau of Vital Statistics, Texas Department of Health, USA	Birth certificate	At birth
Bunin *et al* (1990)	104	Cases diagnosed	1970–1979	(i) Greater DelawareValley Pediatric Tumour Registry, USA (ii) Children's Hospital of Philadelphia Tumor Registry, USA	Parental telephone interview	Preconception, during pregnancy
Wilkins and Hundley (1990)	101	Cases born	1942–1967	Columbus (Ohio) Children's Hospital Tumour Registry, USA	Birth certificate	At birth
Carlsen (1996)	250	Cases born	1943–1980	Hospital records and death certificates, Denmark	Birth certificate	At birth
Kerr *et al* (2000)	183	Cases diagnosed 0–14 years	1976–1987	New York State Cancer Registry, USA	Telephone interview	At birth
Feychting *et al* (2000)	25	Cases born during	1976, 1977, 1981 and 1982	Swedish Cancer Registry, Sweden	Census data	Before conception, during pregnancy
Michaelis *et al* (1996)	80	Cases born in 1988 and diagnosed to 1992		German Childhood Cancer Registry, Germany	Questionnaire and telephone interview	Not stated
Schüz *et al* (2001)	183	Cases diagnosed 0–7 years	1988–1994	German Childhood Cancer Registry, Germany	Questionnaire and telephone interview	Before diagnosis date
Olshan *et al* (1999)	504	Cases diagnosed 0–19 years	1992–1996	Hospitals in the Children's Cancer Group and The Pediatric Oncology Group USA & Canada	Telephone interview	From parental age of 18 years until diagnosis reference date
De Roos *et al* (2001a) De Roos *et al* (2001b) De Roos *et al* (2001c)	504 538 405	Cases diagnosed 0–19 years	1992–1994	Hospitals in the Children's Cancer Group and The Pediatric Oncology Group USA & Canada	Telephone interview	2 year period before the child's date of birth
Pearce *et al* (2006) a Pearce *et al* (2007) a	164	Cases diagnosed 0–24 years	1968–2000	The Northern Region Young Persons’ Malignant Disease Register, The Newcastle Hospital Trust, United Kingdom	Birth certificate	At birth

a Cases from these reports are included in the current study of cases from the National Registry of Childhood Tumours.

**Table 3:** Cases of neuroblastoma: born and diagnosed in Great Britain 1962–1999 by sex and age at diagnosis

	**Cases diagnosed as infants (0 years)**	**Cases diagnosed 1–4 years**	**Cases diagnosed 5–9 years**	**Cases diagnosed 10–14 years**	**Total number of cases in the study**
Male	473	888	210	34	1605
Female	416	720	149	30	1315
Total	889	1608	359	64	**2920**
